# Evaluating COPD care assistance: a comparative study of ChatGPT-3.5, ChatGPT-4.0, and ERNIE bot performance

**DOI:** 10.7189/jogh.16.04297

**Published:** 2026-10-19

**Authors:** Zhongshang Dai, Zihang Zeng, Zijie Zhan, Yan Chen

**Affiliations:** 1 Department of Infectious Diseases, The Second Xiangya Hospital, Changsha, China; 2 FuRong Laboratory, Changsha, China; 3 Clinical Research Centre For Viral Hepatitis In Hunan Province, Hunan, China; 4 Department of Respiratory and Critical Care Medicine, The Second Xiangya Hospital, Changsha, China; 5 Research Unit of Respiratory Disease, Central South University, Changsha, China; 6 Clinical Medical Research Center for Respiratory and Critical Care Medicine in Hunan Province, Changsha, China; 7 Diagnosis and Treatment Center of Respiratory Disease, Central South University, Changsha, China; 8 Department of Radiology, Changde Hospital, Xiangya School of Medicine, Central South University (The First People’s Hospital of Changde City), Changde, China; * Joint first authorship.

**Keywords:** chronic obstructive pulmonary disease, large language model, chatbot, patient education, artificial intelligence, China

## Abstract

**Background:**

Large language model (LLM) chatbots are increasingly used to provide health information, yet their reliability in addressing chronic obstructive pulmonary disease (COPD) care questions in the Chinese context remains unclear. To evaluate and compare the performance of three publicly available LLM chatbots – ChatGPT-3.5, ChatGPT-4.0 (OpenAI), and ERNIE Bot (Baidu) – in answering chronic obstructive pulmonary disease (COPD) care questions in China.

**Methods:**

We performed a cross-sectional comparative evaluation of three publicly available LLM chatbots (ChatGPT-3.5, ChatGPT-4.0, and ERNIE Bot). We developed 40 COPD care questions spanning 7 prespecified domains relevant to patient education and self-management. All questions were submitted in Chinese in independent new chat sessions to minimise context carryover. Three consultant-level respiratory physicians independently rated each response for accuracy (1 = poor, 2 = borderline, 3 = good). We summarised accuracy as the sum of three ratings (range 3–9) and derived a majority-vote accuracy category. For responses rated ‘good’ by majority vote, physicians additionally rated comprehensiveness on a five-point scale. Information quality was further assessed using a modified DISCERN framework and the Patient Education Materials Assessment Tool (PEMAT) understandability and actionability domains.

**Results:**

Most responses were rated as ‘good’ for accuracy across chatbots. ERNIE Bot achieved higher total accuracy scores than ChatGPT-3.5 and ChatGPT-4.0, and a higher proportion of ‘good’ ratings in this Chinese-language evaluation. However, comprehensiveness scores for ‘good’ responses were low across all systems (mean ~ 1.4–2.0/5), and PEMAT actionability was limited, indicating that few responses included concrete, patient-executable steps.

**Conclusions:**

Our findings suggest that LLM chatbots may support basic COPD patient education in Chinese settings, but current responses often lack sufficient comprehensiveness and actionable guidance. These tools should not be used as standalone clinical advice and require appropriate safeguards and clinician oversight.

Chronic obstructive pulmonary disease (COPD) is a common chronic respiratory disease projected to affect nearly 600 million people by 2050, making it a major contributor to the global disease burden and a serious public health threat [1,2]. According to the China Pulmonary Health Study, the prevalence rate of COPD among Chinese adults aged ≥40 has reached as high as 13.7%, and more than one million deaths are attributed to COPD annually [3]. Despite the significant disease burden associated with COPD, there is an exceptionally low level of public awareness and understanding about the condition, irrespective of whether it is in developing or developed nations. Despite its continually rising prevalence in the real world, COPD remains largely overlooked in the public eye [4–7]. This underscores the pressing need to raise awareness of COPD [8].

Meanwhile, patients with COPD who exhibit high compliance are more likely to reap the benefits of treatment, and this compliance is directly linked to their understanding of the disease. Patients who are aware of COPD and have a lower symptom score on the St. George's Respiratory Questionnaire tend to be more adherent to their treatment plan [8]. Research has demonstrated that implementing a COPD self-management educational intervention in offline outpatient clinics can significantly decrease unplanned clinic visits and enhance patients’ quality of life, self-management abilities, and knowledge about the disease [9]. Considering the immense burden placed on outpatient clinics in China, conducting COPD education and management through offline follow-ups is highly impractical.

Open artificial intelligence (AI) chatbots have emerged as promising tools in healthcare, showing strong potential across diverse medical domains such as disease prevention, diagnosis, treatment, patient monitoring, and support [10]. The CONOCEPOC 2019 study in Spain indicates that the most frequent information channel for COPD patients was the media (35.5%) [7], with social networks and the internet also playing a significant role (25.7%) [7]. Compared to traditional search tools, chatbots offer numerous advantages when answering medical questions. Leveraging natural language processing and natural language understanding technologies, chatbots can more accurately comprehend users' intentions and needs, thereby providing personalised medical advice tailored to individual circumstances. The benefits of chatbots in addressing medical queries include immediacy, personalisation, interactivity, emotional support, privacy protection, and resource integration. These advantages position chatbots as having vast application prospects and significant value within the healthcare sector.

Recently, researchers have explored the performance of large language models (LLMs) in the context of COPD, assessing whether chatbots can assist doctors in making COPD-related diagnoses [7,11]. However, the accuracy of chatbot responses to COPD care questions remains uncertain. In this study, we aimed to evaluate and compare the performance of three publicly available LLM chatbots: OpenAI's ChatGPT-3.5, GPT-4.0, and ERNIE Bot, in their ability to respond to queries related to COPD care. We rigorously examined the accuracy and comprehensiveness of each chatbot's responses. Our findings may offer valuable insights into the potential benefits and risks of using information from LLM chatbots to answer common COPD care questions.

## METHODS

### Study design

We conducted a cross-sectional evaluation at the Department of Respiratory and Critical Care Medicine, Second Xiangya Hospital (Changsha, China). We developed 40 COPD care questions to reflect the common demands for patient education and self-management, which were summarised from the WeChat education groups for COPD patients and adapted the seven prespecified domains (*i.e.* definition, pathogenesis, risk factors, clinical presentation, diagnosis, treatment/prevention, prognosis) from previously published literature (Table S1 in the **Online Supplementary Document**) [12]. All questions were submitted in Chinese to each chatbot using the web interface (ChatGPT-3.5, ChatGPT-4.0, and ERNIE Bot) in independent new chat sessions to minimise context carryover. To blind assessors, we exported and formatted the responses as plain text without indicating the generating system.

### Accuracy evaluation

Three respiratory physicians independently scored each response using a three-point rubric: 1 = poor (contains incorrect information likely to mislead or harm), 2 = borderline (minor or possible factual errors unlikely to cause harm), and 3 = good (no apparent factual errors). For each response, we calculated the total accuracy score as the sum of three physician ratings (range 3–9) and a consensus accuracy category (poor/borderline/good) by majority vote (2 of 3). We resolved disagreements by discussion for descriptive reporting and assessed inter-rater reliability on the initial independent ratings.

### Comprehensiveness evaluation

For responses with a majority-vote accuracy rating of ‘good’, physicians further rated comprehensiveness on a five-point scale (*i.e.* 1 = incomplete, 2 = slightly comprehensive, 3 = moderately comprehensive, 4 = comprehensive, 5 = very comprehensive). We summarised comprehensiveness scores across the three raters (*e.g.* median (Mdn) or mean as prespecified) and assessed inter-rater reliability on initial ratings.

### Reliability evaluation

We assessed information quality using an eight-item modified DISCERN framework adapted from the DISCERN [13] instrument to rate reliability and quality of consumer health information, with each item scored from 1 (low) to 5 (high), and the Patient Education Materials Assessment Tool (PEMAT) for understandability (17 items) and actionability (7 items), scored as percentages of applicable items endorsed. Higher scores indicate better understandability/actionability [14].

### Statistical analysis

We summarised continuous outcomes as Mdn and interquartile range (IQR) when non-normally distributed. We used Kruskal-Wallis tests with Dunn’s post-hoc tests and multiplicity adjustment (*e.g.* Holm) for between-chatbot comparisons. We compared proportions of accuracy categories using Pearson χ^2^ tests. In addition to *P*-values, we reported effect sizes (*e.g.* epsilon-squared for Kruskal-Wallis, Cramer’s V for χ^2^) with 95% confidence intervals where applicable.

We used *R*, version 4.1.1 (R Core Team, Vienna, Austria) for all analyses. A two-sided *P* < 0.05 was considered statistically significant.

## RESULTS

The average response length (Chinese character count) was the highest for ERNIE Bot (Mdn = 517; IQR = 454–554), followed by ChatGPT-3.5 (Mdn = 471; IQR = 430–518), and the lowest for ChatGPT-4.0 (Mdn = 369; IQR = 320–422) (**Table 1**).

**Table 1:** Evaluation of AI chatbot responses to COPD-related questions

	ChatGPT3.5	ChatGPT4.0	ERNIE Bot	Test statistics	*P*-value
**Character count**	471 (430–518)	369 (320–422)	517 (454–554)	29.472	<0.001
**mDISCERN**	22.5 (20.5–24)	23 (20.5–25.5)	28.5 (26.5–30)	46.738	<0.001
Poor (<16)	4 (10)	0	0		
Fair (16–31)	36 (90)	40 (100)	38 (95)		
Good (>31)	0	0	2 (5)		
**PEMAT understandability, %**	64.8 (58.8–64.8)	70.6 (58.8–70.6)	64.8 (58.8–64.8)	18.321	<0.001
**PEMAT actionability, %**	0 (0–28.6)	28.6 (14.3–28.6)	28.6 (28.6–28.6)	22.371	<0.001
**Accuracy score**	8 (8–9)	8 (7–9)	9 (8–9)	9.227	0.010
**Inter-rater agreement**					
Kendall’s W	0.392	0.494	0.372		
*P*-value	0.209	0.027	0.286		

AI – artificial intelligence, COPD – chronic obstructive pulmonary disease, PREMAT – Patient Education Materials Assessment Tool

As evaluated by three respiratory specialists, the ERNIE Bot exhibited a significantly higher average total accuracy score (Mdn = 9; IQR = 8–9), outperforming both ChatGPT-3.5 (Mdn = 8; IQR = 8–9, *P* = 0.02) and ChatGPT-4.0 (Mdn = 8; IQR = 7–9, *P* = 0.004) (**Figure 1**).

**Figure 1:**
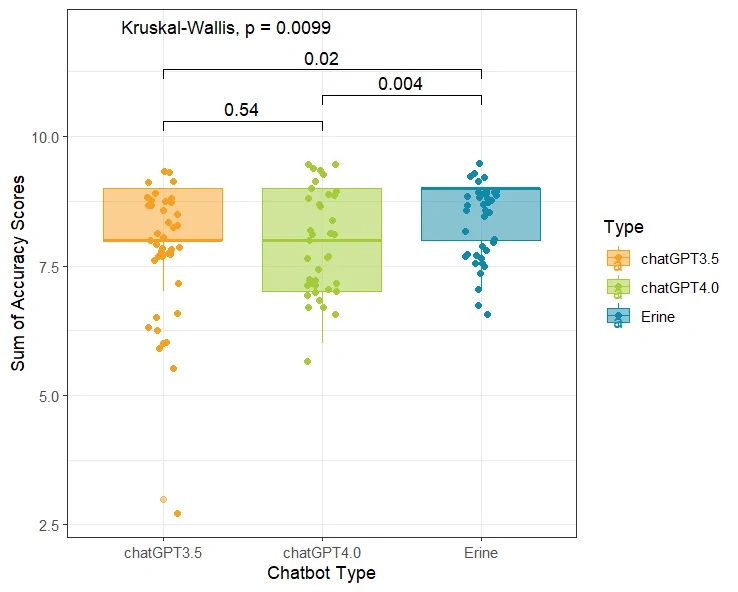
Total accuracy scores of chatbot responses to questions related to COPD. COPD – chronic obstructive pulmonary disease.

Overall, the three systems generated highly accurate responses to questions about COPD. Notably, ChatGPT-3.5 received a ‘poor’ rating. Compared with the other two systems, ChatGPT-4.0s responses were relatively inadequate, mainly because it produced fewer high-quality answers. For the consensus-based accuracy ratings of the three LLM chatbots, ERNIE Bot achieved a ‘good’ rating for 90% of its responses, significantly surpassing 80% for ChatGPT-3.5 and 70% for ChatGPT-4 (**Figure 2**, Panel A–C). Furthermore, ERNIE Bot had a lower proportion of responses with a ‘Borderline’ rating (10%) than ChatGPT-3.5 (17.5%) and ChatGPT-4.0 (30%).

**Figure 2:**
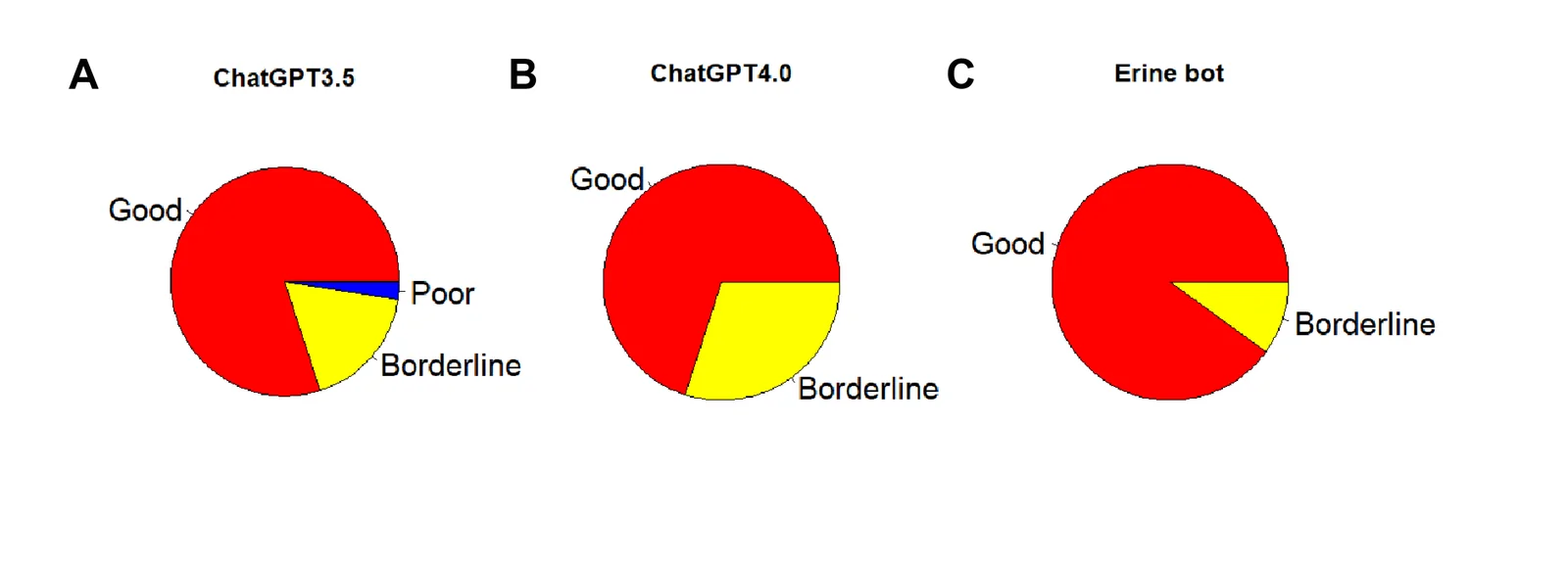
The consensus-based accuracy ratings of the three LLM chatbots. **Panel A.** ChatGPT-3.5. **Panel B.** ChatGPT-4.0. **Panel C.** ERINE Bot. LLM – large language model.

Overall, the three LLM chatbots performed consistently well in the domains of ‘risk factors’ and ‘clinical presentation’, each achieving a 100% ‘good’ rating. In the ‘definition’ domain, the ERNIE Bot achieved a 100% ‘good’ rating, while ChatGPT-3.5 received a ‘poor’ rating for one response (**Table 2**). Nevertheless, ChatGPT-3.5 demonstrated consistent proficiency in the ‘treatment and prevention’ domains, where the ERNIE Bot's performance was comparatively suboptimal. Across most topics, the ERNIE Bot performed better.

**Table 2:** Accuracy domain ratings of AI chatbot responses across seven domains

		ChatGPT3.5			ChatGPT4.0			ERNIE Bot		
	**Questions, n**	**Poor**	**Borderline**	**Good**	**Poor**	**Borderline**	**Good**	**Poor**	**Borderline**	**Good**
**Definition**	4	1	2	1	0	2	2	0	0	4
**Pathogenesis**	3	0	2	1	0	1	2	0	0	3
**Risk factors**	2	0	0	2	0	0	2	0	0	2
**Clinical presentation**	2	0	0	2	0	0	2	0	0	2
**Diagnosis**	4	0	1	3	0	3	1	0	0	4
**Treatment and prevention**	20	0	1	19	0	3	17	0	4	16
**Prognosis**	5	0	1	4	0	3	2	0	0	5

AI – artificial intelligence

None of the three LLM-Chatbots demonstrated optimal performance regarding comprehensiveness scores for ‘good’ rated responses (**Table 3**). ChatGPT-3.5 scored an overall mean score of 1.4, ChatGPT-4.0 scored 1.7, and ERNIE Bot scored 2.2 out of a maximum possible score of 5.

**Table 3:** Comprehensiveness assessment for ‘good’ accuracy rating responses

	n	x̄ (SD)	Mdn
**ChatGPT3.5**	32	1.4 (0.9)	1.3
**ChatGPT4.0**	28	1.5 (1.1)	1.7
**ERNIE Bot**	36	2.0 (0.9)	2.2

Mdn – median, SD – standard deviation, x̄ – mean

We selected the eight core questions from the DISCERN questionnaire to assess the content quality of AI robots as vehicles for health communication. The total DISCERN scores for their responses were 22.5 (range = 20.5–24) for ChatGPT-3.5, 23 (range = 20.5–25.5) for ChatGPT-4.0, and 28.5 (range = 26.5–30) for ERNIE Bot (**Figure 3**). Overall, within this evaluation framework, ERNIE Bot delivered the highest-quality information compared to both ChatGPT-3.5 and ChatGPT-4.0.

**Figure 3:**
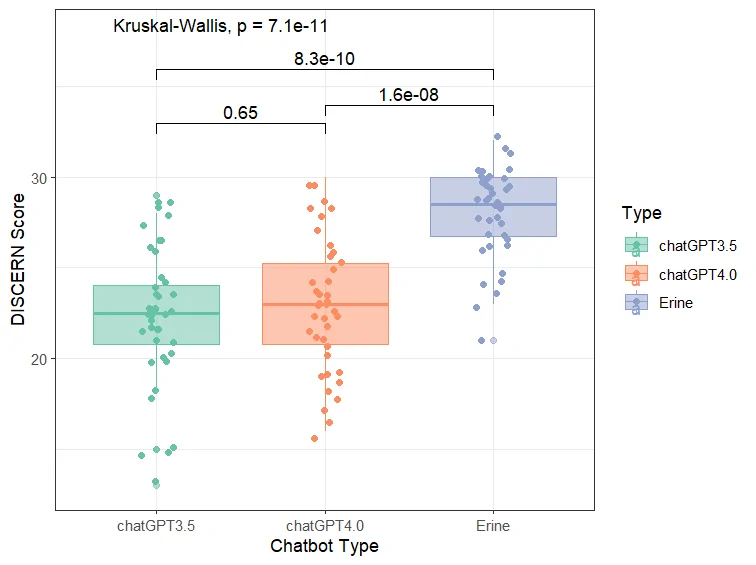
The DISCERN questionnaire to assess the content quality of AI robots. AI – artificial intelligence.

The scores for the PEMAT understandability domain varied. ChatGPT-3.5 scored 58.8–64.8%, ChatGPT-4.0 scored 58.8–70.6%, and ERNIE Bot scored 58.8–64.8% (Figure S1 in the **Online Supplementary Document**). These results suggest a moderate level of understandability across all AI models, with ChatGPT-4.0 performing better than the others.

At the same time, most AI robots show significant deficiencies in the PEMAT actionability domain. The Mdn scores for ChatGPT-3.5 ranged from 0% to 28.6% (Mdn = 0%; IQR = 0–28.6), for ChatGPT-4.0 from 14.3% to 28.6% (Mdn = 28.6%; IQR = 14.3–28.6), and for ERNIE Bot, a consistent score of 28.6% was observed (Figure S2 in the **Online Supplementary Document**).

## DISCUSSION

We comparatively evaluated three LLM chatbots for COPD care questions from the perspective of consultant-level respiratory physicians. Across domains, the systems generally produced accurate information; ERNIE Bot achieved higher accuracy scores in this Chinese-language evaluation, while ChatGPT-4.0 showed more borderline answers in several domains.

Despite acceptable accuracy, all models showed limited comprehensiveness and particularly low PEMAT actionability, suggesting that chatbot outputs often lack concrete, patient-executable steps (*e.g.* when to seek urgent care, how to adjust inhaler technique, or structured self-management actions) [15]. This pattern is consistent with prior evaluations of LLMs in patient education [12], where safety-focused responses may avoid specific instructions and therefore score poorly on actionability.

Several factors may explain ERNIE Bot’s relatively better performance in our setting, including optimisation for Chinese-language queries, greater exposure to Chinese health communication patterns, and system-level alignment for local user contexts [16]. However, model performance is dynamic and may change with updates; therefore, results should be interpreted as a snapshot of the systems tested at the time of evaluation.

### Clinical and public health implications

Overall, LLM chatbots may be useful for basic COPD education and awareness raising, especially where clinician time is limited, but they should not replace professional medical advice. To reduce the risk of misinformation, chatbots should be used with safeguards such as clear disclaimers, links to guideline-based resources, and clinician review for high-stakes decisions [17].

We did not fully explore the limitations of chatbots when dealing with complex and personalised medical issues, nor did we conduct an in-depth study of their long-term effects [18]. Issues such as whether chatbots can consistently provide accurate and useful information, and the long-term changes in patients' trust and satisfaction towards them, warrant further exploration. Additionally, we may have overlooked the ethical and legal challenges that chatbots may encounter in practical applications [19,20]. For instance, ensuring that the information provided by chatbots complies with medical ethics standards and handling potential disputes arising from chatbot recommendations within the legal framework are crucial considerations. Furthermore, we may have inadequately assessed the applicability and acceptability of chatbots among different patient groups, as patients of various age groups, cultural backgrounds, and educational levels may have differing perspectives and experiences regarding the use and effectiveness of chatbots. In summary, our research has certain shortcomings and limitations when exploring the application of chatbots in the medical field [18]. Future research should further expand and deepen these aspects to more comprehensively evaluate the actual effects and potential value of chatbots.

## CONCLUSIONS

Our results suggest that LLM chatbots may support basic COPD patient education in Chinese settings, but current responses often lack sufficient comprehensiveness and actionable guidance. These tools should not be used as standalone clinical advice and require appropriate safeguards and clinician oversight.

## Additional material


Online Supplementary Document

## Data Availability

**Data availability:** Data are available from the corresponding author upon reasonable request.
