# Supplementary material for: Evaluating COPD care assistance: a comparative study of ChatGPT-3.5, ChatGPT-4.0, and ERNIE bot performance
Source: J Glob Health. 2026 Oct 19;16:04297. doi: 10.7189/jogh.16.04297 (PMC13679348; doi:10.7189/jogh.16.04297)
Supplement: Supplementary file 1 [file jogh-16-04297-s001.pdf]

Supplement to: Dai Z, Zeng Z, Zheng C, Zhan Z, Chen Y. Evaluating COPD care assistance: a comparative study of ChatGPT-3.5, ChatGPT-4.0, and ERNIE bot performance. J Glob Health. 2026;16:04297.

Benchmarking large language models' performances for COPD: a comparative analysis of ChatGPT-3.5, ChatGPT-4.0, and Ernie Bot

Topics definition, pathogenesis, risk factors, clinical presentation, diagnosis, treatment and prevention, and prognosis.

Questions - Definition

1. What is COPD?
2. What is the global prevalence of COPD?
3. Is the incidence of COPD related to age?
4. What is the current social burden caused by COPD?

Questions - Pathogenesis

1. At what age does COPD commonly develop?
2. What causes COPD?
3. How cigarette smoke causes the onset of COPD?

Questions - Risk factors

1. What puts my parent at risk of COPD?
2. Is COPD hereditary? My parent has COPD, how likely is it for me to get COPD?

Questions - Clinical presentation

1.What are signs and symptoms that suggests my parent has COPD?

2.What symptoms indicate that COPD is constantly worsening?

Questions - Diagnosis

1. How is COPD diagnosed?
2. What should I do if I suspect my parent has COPD?
3. What tests can my parents do to clarify the diagnosis of COPD?
4. Is it safe for elderly people to have lung function tests?

Questions - Treatment and prevention

1. What can I do to prevent COPD?
2. My parent has COPD, what are the available treatments to slow down the progression of COPD?
3. How can I prevent COPD/COPD progression without the use of medications?
4. What are the inhalation of drugs available to prevent COPD/COPD progression?
5. How effective is inhaled corticosteroids at helping to prevent COPD/COPD progression?
6. How does inhaled corticosteroids help to prevent COPD/COPD progression?
7. Is it safe for my parents to use inhaled corticosteroids at such an old age?
8. How effective is increasing cardiorespiratory exercise at helping to prevent COPD/COPD progression?

9. How effective is reducing smoking at helping to prevent COPD/COPD progression?

10. What is the maximum duration of smoking time per day before my parent is at risk of COPD/COPD progression?

11. How much cease-smoking time does my parent require to prevent COPD/COPD progression?

12. What type of diet can help to prevent COPD/COPD progression?

13. Are there any available breathing exercises that can help to prevent COPD/COPD progression?

14. What medications are available to prevent COPD/COPD progression?

15. Is long-acting inhaled bronchodilator safe in elderly people?

16. What are the side effects of inhaled corticosteroids/ long-acting inhaled bronchodilator?

17. How effective is long-acting inhaled bronchodilator at helping to prevent COPD/COPD progression?

18. What are the types of long-acting inhaled bronchodilators, and which is the most effective?

19. How does long-acting inhaled bronchodilator help to prevent COPD/COPD progression?

20. My parent has not developed COPD, should he/ she start using long-acting inhaled bronchodilator?

21. My parent is on inhaled corticosteroids/ long-acting inhaled bronchodilator for COPD control, when can it be stopped?

Questions - Prognosis

1. Is COPD reversible?

2. Why is COPD a concern? What are the potential complications of COPD?

3. Can COPD cause respiratory failure?

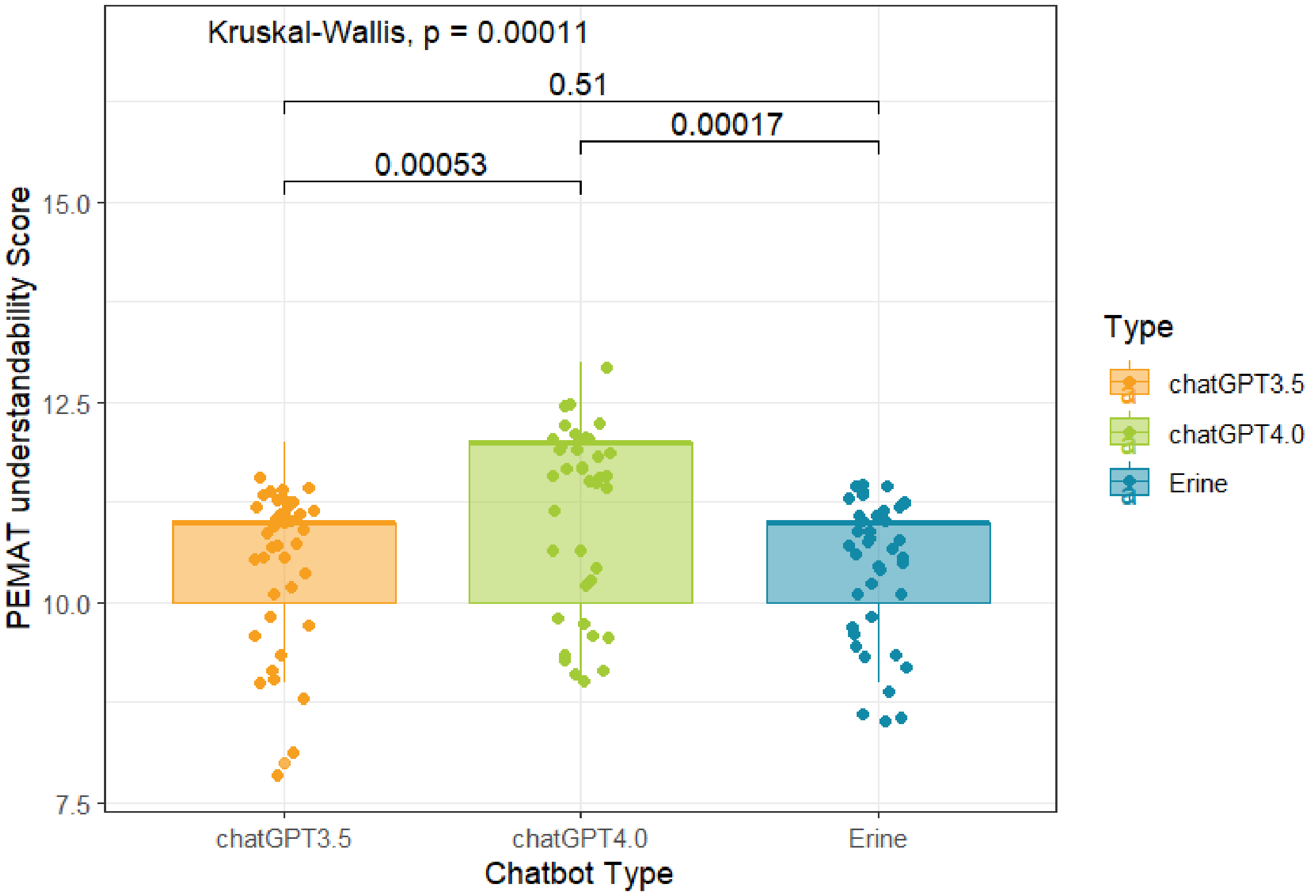

Figure S1. The scores for the PEMAT understandability.

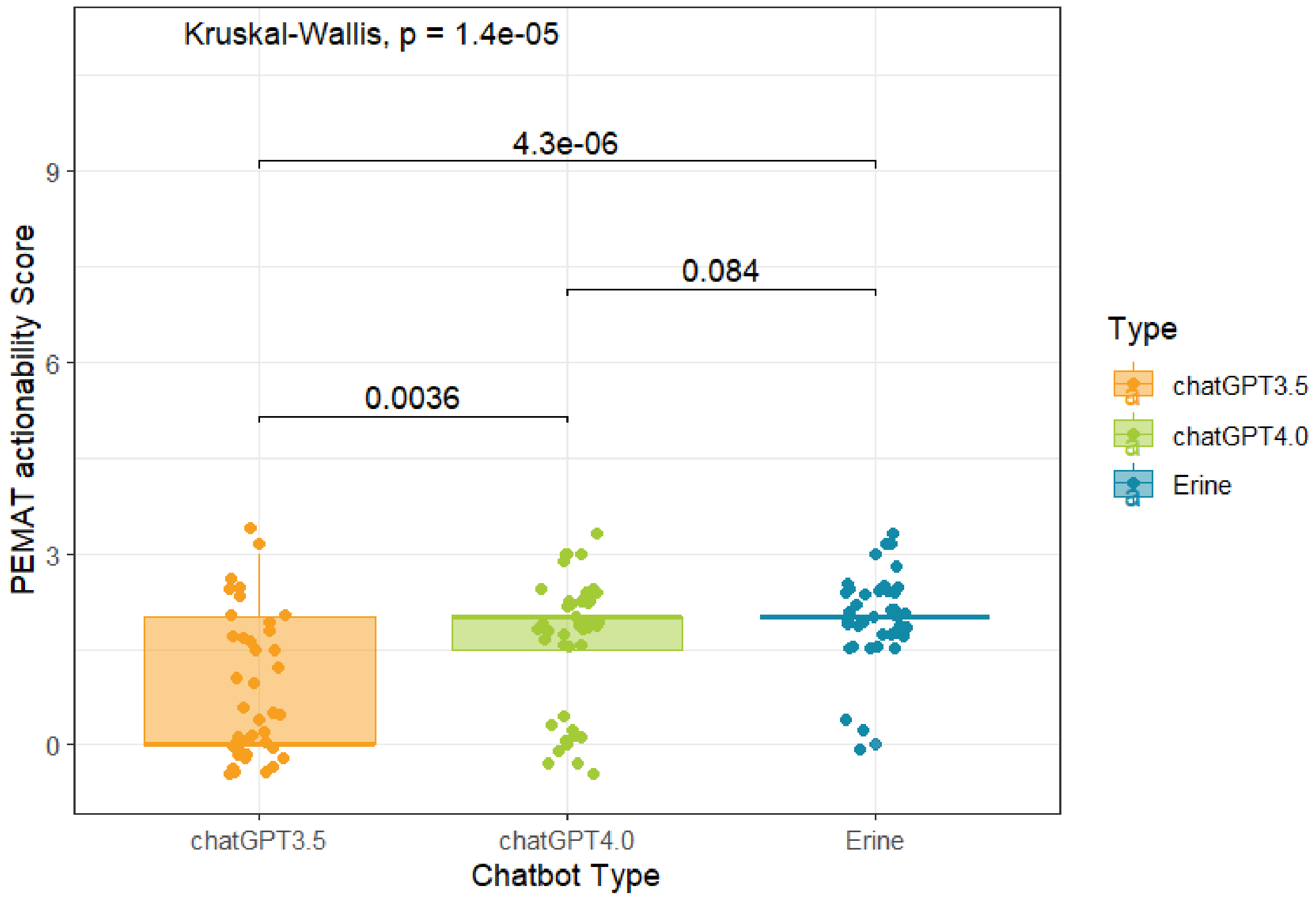

Figure S2. The scores for the PEMAT actionability.
